# Restoration of Responsiveness of Phospholipase Cγ2-Deficient Platelets by Enforced Expression of Phospholipase Cγ1

**DOI:** 10.1371/journal.pone.0119739

**Published:** 2015-03-20

**Authors:** Yongwei Zheng, Tamara Adams, Huiying Zhi, Mei Yu, Renren Wen, Peter J. Newman, Demin Wang, Debra K. Newman

**Affiliations:** 1 Blood Research Institute, BloodCenter of Wisconsin, Milwaukee, Wisconsin, United States of America; 2 Key Laboratory of Developmental Genes and Human Disease, Ministry of Education, Institute of Life Science, Southeast University, Nanjing, Jiangsu, People’s Republic of China; 3 Department of Pharmacology and Toxicology, Medical College of Wisconsin, Milwaukee, Wisconsin, United States of America; 4 Department of Cell Biology, Neurobiology and Anatomy, Medical College of Wisconsin, Milwaukee, Wisconsin, United States of America; 5 Department of Microbiology and Molecular Genetics, Medical College of Wisconsin, Milwaukee, Wisconsin, United States of America; National Cerebral and Cardiovascular Center, JAPAN

## Abstract

Receptor-mediated platelet activation requires phospholipase C (PLC) activity to elevate intracellular calcium and induce actin cytoskeleton reorganization. PLCs are classified into structurally distinct β, γ, δ, ε, ζ, and η isoforms. There are two PLCγ isoforms (PLCγ1, PLCγ2), which are critical for activation by tyrosine kinase-dependent receptors. Platelets express both PLCγ1 and PLCγ2. Although PLCγ2 has been shown to play a dominant role in platelet activation, the extent to which PLCγ1 contributes has not been evaluated. To ascertain the relative contributions of PLCγ1 and PLCγ2 to platelet activation, we generated conditionally PLCγ1-deficient, wild-type (WT), PLCγ2-deficient, and PLCγ1/PLCγ2 double-deficient mice and measured the ability of platelets to respond to different agonists. We found that PLCγ2 deficiency abrogated αIIbβ3-dependent platelet spreading, GPVI-dependent platelet aggregation, and thrombus formation on collagen-coated surfaces under shear conditions, which is dependent on both GPVI and αIIbβ3. Addition of exogenous ADP overcame defective spreading of PLCγ2-deficient platelets on immobilized fibrinogen, suggesting that PLCγ2 is required for granule secretion in response to αIIbβ3 ligation. Consistently, αIIbβ3-mediated release of granule contents was impaired in the absence of PLCγ2. In contrast, PLCγ1-deficient platelets spread and released granule contents normally on fibrinogen, exhibited normal levels of GPVI-dependent aggregation, and formed thrombi normally on collagen-coated surfaces. Interestingly, enforced expression of PLCγ1 fully restored GPVI-dependent aggregation and αIIbβ3-dependent spreading of PLCγ2-deficient platelets. We conclude that platelet activation through GPVI and αIIbβ3 utilizes PLCγ2 because PLCγ1 levels are insufficient to support responsiveness, but that PLCγ1 can restore responsiveness if expressed at levels normally achieved by PLCγ2.

## Introduction

**Fig 1:**
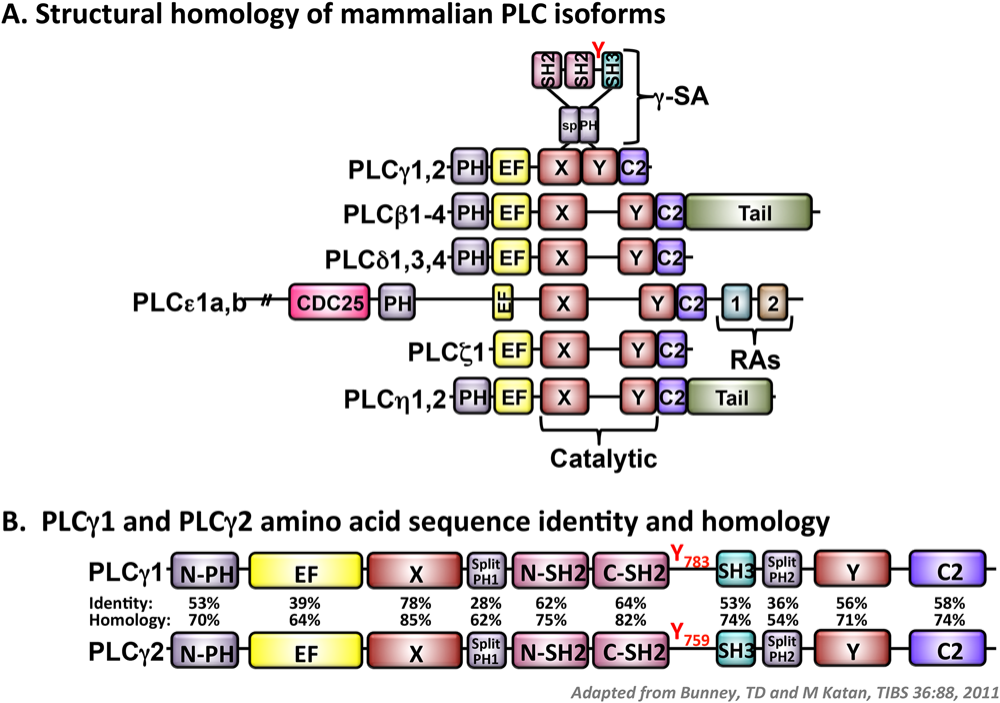
Key structural differences between PLC isoforms. (A) Domain organization of PLC family members, all of which possess an N-terminal pleckstrin homology (PH) domain (light purple), EF hands (yellow), split catalytic X and Y domains (red), and a C-terminal C2 domain (dark purple). PLCγ isoforms possess a γ-specific array (γ-SA) that encompasses a split PH domain, two Src homology (SH) 2 domains (pink), one SH3 domain (teal) and an activating tyrosine (Y) phosphorylation site. PLCβ and PLCη family members possess unique C-terminal tails (green). PLCε possesses a unique N-terminal CDC25 domain (pink) and two Ras-association (RA) domains (teal and brown) at the C-terminus. Schematic is adapted from reference #7. (B) Degree of amino acid sequence identity and homology for each domain of PLCγ1 and PLCγ2.

PLC-mediated hydrolysis of plasma membrane phosphatidylinositol 4,5-bisphosphate (PIP_2_) to generate inositol 1,4,5-trisphosphate (IP_3_) and diacylglycerol (DAG) is critical for receptor-mediated cellular activation.[1] IP_3_ triggers Ca^2+^ mobilization by binding to its receptor on the endoplasmic reticulum in nucleated cells or on the dense tubular system in platelets.[2, 3] DAG is responsible for activation of protein kinase C (PKC), which can further stimulate cytoskeletal rearrangements.[4, 5] As shown in Fig. 1A, mammalian PLCs are grouped on the basis of structure into six different isoforms, including PLCβ, γ, δ, ε, ζ and η.[6, 7] All PLC isoforms contain two highly conserved regions, referred to as X and Y, which together comprise the catalytic domain responsible for generation of the secondary messengers IP_3_ and DAG.[8] PLCγ isoforms have unique features that distinguish them from other PLC isoforms. These include two Src homology 2 (SH2) domains and one Src homology 3 (SH3) domain, which are localized between the X and Y catalytic regions and which mediate associations with effector molecules that contain phosphorylated tyrosine residues and proline-rich sequences, respectively.[8–10] There are two members of the PLCγ family, including PLCγ1 and PLCγ2 (Fig. 1B). Whereas PLCγ1 is expressed ubiquitously, PLCγ2 expression is limited to cells of the hematopoietic lineage.[1] Both PLCγ1 and PLCγ2 function downstream of immune and adhesion receptors that are coupled to immunoreceptor tyrosine-based activation motif (ITAM)-containing subunits. In addition, PLCγ1 functions downstream of receptor tyrosine kinases, such as fibroblast growth factor receptor (FGF-R) and platelet-derived growth factor receptor (PDGF-R).[11] Homozygous disruption of the PLCγ1 gene in mice results in lethality at embryonic day 9,[12] indicating that PLCγ1 plays an essential role in cell growth, differentiation and development. Deficiency of PLCγ2 in mice does not cause embryonic lethality; however, PLCγ2-deficient mice exhibit abnormalities in B cell development and function, separation of blood from lymphatic vessels, and platelet function.[13, 14]. PLCγ1 and PLCγ2 are expressed at different levels in different cell types and at different stages of development. Consequently, the extent to which these enzymes are capable of functioning redundantly cannot be determined from studies of knockout mice that fail to express one or the other isoform.

Platelets possess four major tyrosine kinase-dependent receptors capable of inducing shape change, granule secretion and aggregation following ligand binding. These include the glycoprotein (GP)Ib-IX-V receptor for von Willebrand factor, the GPVI receptor for collagen and laminin, the integrin αIIbβ3, which serves as the platelet-specific receptor for fibrinogen and CLEC-2, which is the receptor for podoplanin on lymphatic endothelium and the snake venom rhodocytin. Engagement of these receptors results in a series of tyrosine phosphorylation events that culminates in activation of PLCγ, generation of IP_3_ and DAG, and platelet responsiveness.[15, 16] The role of PLCγ2 in these processes has been evaluated by studying PLCγ2-deficient (PLCγ2^-/-^) mice, which exhibited a prolonged bleeding time [17] and defective thrombus formation following laser injury of mesenteric arterioles *in vivo* [18], and in which GPVI- and CLEC-2-dependent platelet responses were abolished.[17, 19–21] These findings established that PLCγ2 is indispensable for GPVI-mediated platelet activation. With respect to αIIbβ3-mediated platelet responses, formation of filopodia and lamellipodia by mouse platelets on fibrinogen-coated surfaces was dramatically inhibited in the absence of PLCγ2 and, consistent with these morphological defects, PLCγ2^-/-^ platelets exhibited minimal calcium flux and phosphatidic acid production following adhesion to fibrinogen.[22, 23] Furthermore, relative to platelets from wild-type mice, platelets from PLCγ2-deficient mice formed less stable thrombi on fibrinogen under flow conditions and were impaired in their ability to retract a fibrin clot. [22, 24] These findings suggest that PLCγ2 plays an important, but not indispensable, role in αIIbβ3-mediated platelet activation, and raise the possibility that the low levels of PLCγ1 that are present in platelets are able to support platelet activation by αIIbβ3.

The embryonic lethality of PLCγ1-deficient mice has heretofore precluded assessment of the role of PLCγ1 in platelet function.[12] However, a mouse model that allows for conditional deletion of the PLCγ1 gene has recently been generated.[25] In the present study, we used conditionally PLCγ1- and PLCγ1/γ2-deficient mice, along with a retrovirus-mediated gene transfer and bone marrow reconstitution strategy, to dissect the extent to which PLCγ1 contributes to platelet activation. We found that platelet activation by the tyrosine kinase-dependent adhesion receptors GPVI and αIIbβ3 normally requires PLCγ2 because the levels at which PLCγ1 is expressed are limiting. If over-expressed, however, PLCγ1 can fully support platelet activation by these receptors. The full functional redundancy of PLCγ1 and PLCγ2 that is characteristic of platelets is unique amongst hematopoietic cells.

## Materials and Methods

### Mice

PLCγ1-floxed mice (PLCγ1^fl/fl^) and PLCγ2^-/-^ mice on a C57BL/6 genetic background have been previously described.[14, 25] To generate PLCγ1^fl/fl^ Mx1Cre or PLCγ1^fl/fl^ PLCγ2^-/-^ Mx1Cre mice, PLCγ1^fl/+^ or PLCγ1^fl/+^ PLCγ2^+/-^ mice were bred with Mx1Cre mice (Jackson Laboratory stock 005673). To induce the expression of Cre recombinase, 8–10 week old PLCγ1^+/+^Mx1Cre, PLCγ1^fl/fl^Mx1Cre, PLCγ2^-/-^Mx1Cre and PLCγ1^fl/fl^PLCγ2^-/-^Mx1Cre mice were administered intraperitoneal injections of 0.3 mg of poly(I:C) (Amersham) twice at 2-day intervals. To generate bone marrow chimeric mice, bone marrow cells from these mice were harvested two weeks after poly(I:C) treatment and injected into lethally irradiated (1100 rads) 8-week old C57BL/6 CD45.1 congenic mice (Jackson Laboratory stock 002014). Eight weeks after bone marrow transplantation, chimeric mice were used for platelet experiments. Mice were maintained in the Biological Resource Center at the Medical College of Wisconsin (MCW). All animal protocols were approved by the MCW Institutional Animal Care and Use Committee.

### Antibodies and reagents

Antibodies specific for Syk (N-19 #sc-1077), PLCγ1 (1249, #sc-81) and PLCγ2 (Q-20, #sc-407) were purchased from Santa Cruz Biotechnology. The anti-FLAG antibody (M2, #A8592) and TRITC-conjugated phalloidin (#77418) were purchased from Sigma Aldrich. Collagen for platelet aggregation was purchased from Chrono-Log Corporation. Thrombin receptor activating peptide (TRAP; amino acid sequence SFLLRN) was synthesized by the Protein Chemistry Core Laboratory at the Blood Research Institute of BloodCenter of Wisconsin.

### Expression of recombinant truncated PLCγ1 and PLCγ2 proteins in COS-7 cells

COS-7 cells were transfected with rPLCγ1ΔPHnFL-PRK5 or rPLCγ2ΔPH-EFnFL-PRK5 plasmids (0.5 μg plasmid/10^5^ cells). After 48 hours, transfected cells were lysed in 500 μl cell lysis buffer (20 mM TrisHCl, 50 mM NaCl, 5 mM EDTA, 1% Triton-100, 3 μg/ml aprotinin, 2 μg/ml pepstatin A, 1 μg/ml leupeptin) for 30 min on ice. Lysates were mixed with an equal volume of 2X SDS loading buffer, boiled for 5 min, separated by SDS-PAGE, and subjected to Western blot analysis.

### Preparation of washed platelets

Mouse blood was drawn from the inferior vena cava of anesthetized mice into a syringe containing 3.8% sodium citrate (1/10 volume), then diluted 1:1 with Tyrode’s buffer (137 mM NaCl, 13.8 mM NaHCO_3_, 2.5 mM KCl, 0.36 mM NaH_2_PO_4_, 20 mM HEPES, and 0.1% glucose). Diluted whole blood was supplemented with 50 ng/ml prostaglandin E1 (PGE_1_) and spun at 200g for 8 minutes at room temperature without brakes. Platelet-rich plasma (PRP) was collected and, after the addition of 50 ng/ml PGE_1_, platelets were pelleted at 800g for 8 minutes. Platelets were washed in Tyrode’s buffer containing 50 ng/ml PGE_1_ and 1 mM EDTA and spun at 800g for 8 minutes. Washed platelets were finally resuspended in Tyrode’s buffer to the indicated final concentration.

Highly purified platelets were obtained by depleting washed mouse platelets, prepared as described above, of contaminating leukocytes and erythrocytes. Briefly, 10 μl each of anti-CD45 and anti-Ter-119 Microbeads (Miltenyi) were added to washed mouse platelets (10^7^ platelets/90 μl) and allowed to incubate at 4°C for 15 minutes, after which 2 ml of Miltenyi Buffer 1 was added and the suspension was centrifuged at 300 g for 10 min. The supernatant was completely removed and the pelleted platelets and microbeads were suspended in Buffer 1. An LS Column (Miltenyi) was placed in a MACS Separator magnetic field and rinsed with 3 ml of Buffer 1 (1x PBS with 1% BSA), after which the platelet/microbead suspension was applied to the column. Platelets, to which anti-CD45 and anti-Ter-119 do not bind, were collected in the effluent. The column was washed with 3 times with 3 ml of Buffer 1 and the total effluent was collected. Flow cytometry was used to confirm the absence T cells, B cells, and monocytes in the highly purified platelet population (data not shown). Highly purified platelets were lysed in an equal volume of 2x lysis buffer. Undiluted and 1:70 diluted platelet lysates were used for Western blot analysis of PLCγ1 and PLCγ2 expression levels, respectively.

### Immunoblot analysis

For biochemical analyses, washed platelets were lysed directly with 2X immunoprecipitation (IP) buffer (300 mM NaCl, 20mM Tris, 10 mM EDTA, 2 mM Na_3_VO_4_, 2% NP40; pH7.6) containing 2% protease inhibitor (Thermo Scientific) and phosphatase inhibitor (EMD Millipore) cocktails. Platelet lysates were subjected to SDS-polyacrylamide gel electrophoresis and immunoblot analysis. Tyrosine kinase Syk was chosen as a loading control in the immunoblot analysis, as Syk is highly expressed in platelets and plays a key role in platelet signal transduction.

### Collagen-induced platelet aggregation

Platelet aggregation assays were performed using a lumi-aggregometer (Chrono-Log). Washed platelets (300 μl) at a concentration of 1×10^8^/ml in Tyrode’s buffer containing 1 mM CaCl_2_ were added to a siliconized glass cuvette and stirred at 1000 rpm for 30 seconds at 37°C. Platelet activation was initiated by addition of 6 μg/ml or 50 μg/ml collagen. After allowing platelets to aggregate in response to collagen for 5 minutes, TRAP (5 μg/ml) was added to the same cuvette as a positive control.

### *In vitro* thrombus formation under flow conditions

Thrombus formation was evaluated by perfusing whole blood over collagen-coated micro-channels under arterial shear conditions. Briefly, Vena8 FLUORO+ Biochips (Cellix Ltd) were coated overnight at 4°C with fibrillar collagen (50 μg/ml) and blocked with Hank’s Balancing Salt Solution containing 0.1% BSA. Whole blood from the various mice to be tested was anti-coagulated with heparin and PPACK, labeled with mepacrine (CalBiochem), and perfused over collagen-coated micro-channels at a shear rate of 1333s^-1^. Images of platelet adhesion and thrombus formation were acquired by epifluorescence microscopy in real time at a frame rate of one frame per second. Quantification of thrombus formation is reported as the mean integrated fluorescence intensity (IFI) per μm^2^. Image analysis was performed using Metamorph software (Universal Imaging).

### Platelet spreading on immobilized fibrinogen

Eight-chamber glass tissue-culture slides (Becton Dickinson) were coated with 3 μg/ml fibrinogen (Fg) or 1% bovine serum albumin (BSA) that had been pre-cleared of IgG using protein G beads in PBS at 4°C overnight. Wells were blocked with 1% BSA for 1 hour at room temperature prior to cell spreading. Washed platelets (200 μl) at a concentration of 7.5×10^6^/ml in Tyrode’s buffer supplemented with 1 mM CaCl_2_ and 2 mM MgCl_2_ were allowed to spread on immobilized Fg or BSA for the indicated periods of time at 37°C. In some cases, ADP (Bio/DATA Corporation) was added at a final concentration of 20 μm. Non-adherent platelets were removed by washing slides with 37°C PBS 3 times. The remaining adherent platelets were fixed with 3% paraformaldehyde/PBS for 30 minutes and permeabilized for 5 minutes at room temperature with 0.5% NP40/PBS. Slides containing adherent platelets were blocked with 3% BSA at room temperature for 1 hour and then stained with phalloidin-TRITC (1 μg/ml) at 4°C overnight. Samples were mounted in Vectashield mounting medium (Vector Laboratories). Images were acquired with a Photometrics SenSys camera (Photometrics) using a Zeiss Axioscop microscope (Carl Zeiss) with a Zeiss 60 x lens (0.7 numeric aperture) and analyzed using Metamorph software (Universal Imaging). Results are reported as the mean area of spread platelets (μm^2^/platelet on immobilized Fg—μm^2^/platelet on BSA) and the mean percent of platelet spreading (number of spread platelets/total number of platelets x 100), where spread platelets were defined as those with pseudopodia.[22]

### Platelet factor 4 (PF4) ELISA

Washed platelets (200 μl) at a concentration of 7.5×10^6^/ml were allowed to spread on 8-chamber glass tissue-culture slides coated with 3 μg/ml Fg or 1% BSA for 1 hour at 37°C. Supernatants were collected and spun at 800 g for 5 minutes. The concentration of PF4 in each sample as determined using the Mouse CXCL4/PF4 Quantikine ELISA kit (R&D systems).

### Retroviral Transduction and Bone Marrow Transplantation

Retroviral transduction and bone marrow transplantation were performed as previously described.[26] Briefly, the rat PLCγ1 or rat PLCγ2 gene was cloned into a bicistronic retrovirus MSCV-IRES-GFP vector, in which expression of the cloned gene and green fluorescent protein (GFP) is under the control of the murine stem cell virus promoter. GFP fluorescence is used as a marker for identification of retrovirally transduced cells. Conditioned media containing high-titer, amphotropic retrovirus particles were derived by cotransfection of 293T cells with the retrovirus vector expressing the cloned gene and GFP and with a pEQPAM3 helper plasmid containing the required gag, pol, and env retroviral genes. This media was used to transduce ecotropic packaging cells (GP+E86) with 6 μg/ml polybrene (Sigma). Cells exhibiting high GFP expression were sorted and subsequently expanded as virus-producing cells. Mouse bone marrow cells were transduced with retrovirus as follows: PLCγ1/γ2 double-deficient mice (8 to 12 weeks old) were injected intraperitoneally with 150 mg/kg of 5-fluorouracil 48 hours before bone marrow harvest. Bone marrow cells were isolated and prestimulated with 20 ng/ml of IL3, 50 ng/ml of IL6 and 50 ng/ml stem cell factor (SCF) for 48 hours. Cells were then co-cultured on irradiated ecotropic producer cells (GP+E86) in the presence of IL3, IL6, SCF and polybrene (6 μg/ml). After 48 hours, 1 to 2 x 10^6^ bone marrow cells were introduced via tail veil injection into lethally irradiated (1100 rads) 8-week old C57BL/6 wild-type mice (Jackson Laboratory stock 000664). Eight weeks later, mice were used for platelet studies.

### Statistical analysis

Statistically significant differences were identified by performing a one-way ANOVA followed by a two-tailed unpaired Student’s t test using Graphpad Prism 6.0 software.

### Ethics Statement

Mice were maintained in a facility free of well-defined pathogens under the supervision of the Biological Resource Center at the Medical College of Wisconsin. All animal protocols were approved by the Institutional Animal Care and Use Committee of the Medical College of Wisconsin (Protocols #AUA00000952 and AUA00000929). For the experiments reported in this manuscript, mice were anesthetized with a lethal injection of a mixture of ketamine and xylazine or with isoflurane inhalation. Blood was drawn from the inferior vena cava and organs and tissues were removed from unconscious mice, after which mice were euthanized by cervical dislocation or carbon dioxide inhalation.

## Results

### PLCγ2 is 400X more abundant than PLCγ1 in murine platelets

**Fig 2:**
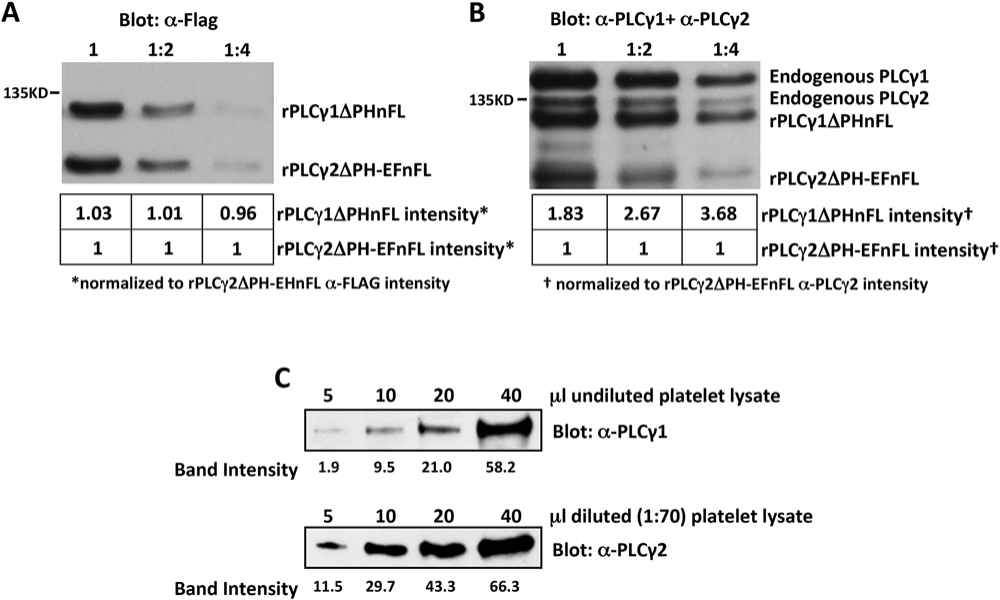
Quantification of relative levels of PLCγ1 and PLCγ2 in mouse platelets. (A and B) Lysates were prepared from COS-7 cells transfected with a plasmid encoding a FLAG-tagged form of either PLCγ1 in which the pleckstrin homology domain was deleted (rPLCγ1ΔPHnFL) or PLCγ2 in which the PH domain and EF hands were deleted (rPLCγ2ΔPH-EFnFL) and mixed. (A) Serial two-fold dilutions of the mixed COS-7 lysates were subjected to Western blot analysis with antibodies specific for the FLAG tag. Numbers under each lane indicate the densities of the rPLCγ1ΔPHnFL and rPLCγ2ΔPH-EFnFL bands relative to that of the rPLCγ2ΔPH-EFnFL band, which was assigned an arbitrary value of 1. These data demonstrate that rPLCγ1ΔPHnFL and rPLCγ2ΔPH-EFnFL proteins were equally loaded in each lane. (B) The same serial dilutions of mixed COS-7 cell lysates were subjected to Western blot analysis with a mixture of PLCγ1- and PLCγ2-specific antibodies. Note that endogenous PLCγ1 and PLCγ2 are also detected by these antibodies. Numbers under each lane indicate the density of the rPLCγ1ΔPHnFL and rPLCγ2ΔPH-EFnFL bands relative to that of the rPLCγ2ΔPH-EFnFL band, which was assigned an arbitrary value of 1. These data demonstrate that the anti-PLCγ1 antibody recognizes PLCγ1 about three times better than the anti-PLCγ2 antibody recognizes PLCγ2. (C) Increasing amounts of undiluted (top) or 1:70 diluted (bottom) highly purified mouse platelet lysate were subjected to Western blot analysis with antibodies specific for PLCγ1 (top) or PLCγ2 (bottom). Numbers under each lane indicate the density of each band. Note that approximately 140X more platelet lysate was required to achieve a PLCγ1 band intensity equivalent to that of PLCγ2. Together with the finding that anti-PLCγ1 recognizes PLCγ1 approximately 3X better than anti-PLCγ2 recognizes PLCγ2, we conclude that mouse platelets have ~400X less PLCγ1 than PLCγ2.

Platelets have been reported to express up to four times more PLCγ2 than PLCγ1 at the transcript level [27]; however, the relative abundance of PLCγ1 and PLCγ2 protein has not yet been determined. We used a quantitative Western blotting strategy to determine the relative levels of expression of PLCγ1 and PLCγ2 protein in mouse platelets. We first determined the relative affinities of Western blotting PLCγ1- and PLCγ2-specific antibodies for their respective targets. To accomplish this, a 130 kDa N-terminally flag-tagged mutant form of rat PLCγ1, in which the pleckstrin homology (PH) domain was deleted (rPLCγ1ΔPHnFL), and a 107 kDa flag-tagged mutant form of rat PLCγ2, in which both the PH and EF domains were deleted (rPLCγ2ΔPH-EFnFL), were over-expressed separately in COS-7 cells. Transfected COS-7 cell lysates with equal amounts of rPLCγ1ΔPHnFL and rPLCγ2ΔPH-EFnFL were mixed, serially diluted and subjected to Western blot analysis with antibodies specific for the flag tag to confirm equal loading of the two proteins (Fig. 2A). The same samples were then subjected to Western blot analysis using a mixture of antibodies specific for PLCγ1 or PLCγ2, each of which binds to the C-terminus of its target (and therefore is not affected by the PH or PH-EF deletion) and does not cross-react with the other isoform. Densitometric analysis of the PLCγ1/PLCγ2 blots of COS-7 cell lysates revealed that the PLCγ1-specific antibody recognized rPLCγ1ΔPHnFL an average of ~3 times better than the PLCγ2-specific antibody recognized rPLCγ2ΔPH-EFnFL (Fig. 2B). To quantify the relative levels of PLCγ1 and PLCγ2 protein in platelets, increasing amounts of undiluted or 1:70 diluted lysates of highly purified mouse platelets were subjected to Western blot analysis with antibodies specific for PLCγ1 or PLCγ2, respectively. As shown in Fig. 2C, densitometric analysis of the PLCγ1/PLCγ2 blots of platelet lysates revealed that approximately 140 times more platelet lysate was required to achieve a PLCγ1 band intensity equivalent to that of PLCγ2 (e.g., the PLCγ1 band intensity in 10 μl of undiluted platelet lysate was equivalent to the PLCγ2 band intensity observed with 5 μl of 1:70 diluted platelet lysate). Together with the finding that anti-PLCγ1 recognizes PLCγ1 approximately 3 times better than anti-PLCγ2 recognizes PLCγ2, we conclude that mouse platelets have ~400X less PLCγ1 than PLCγ2 (~140X more lysate required for equivalent density of PLCγ1 relative to PLCγ2 bands x ~3X better recognition of PLCγ1 than PLCγ2).

### Collagen-induced platelet activation and thrombus formation are severely impaired in the absence of PLCγ2 but unaffected by the absence of PLCγ1

**Fig 3:**
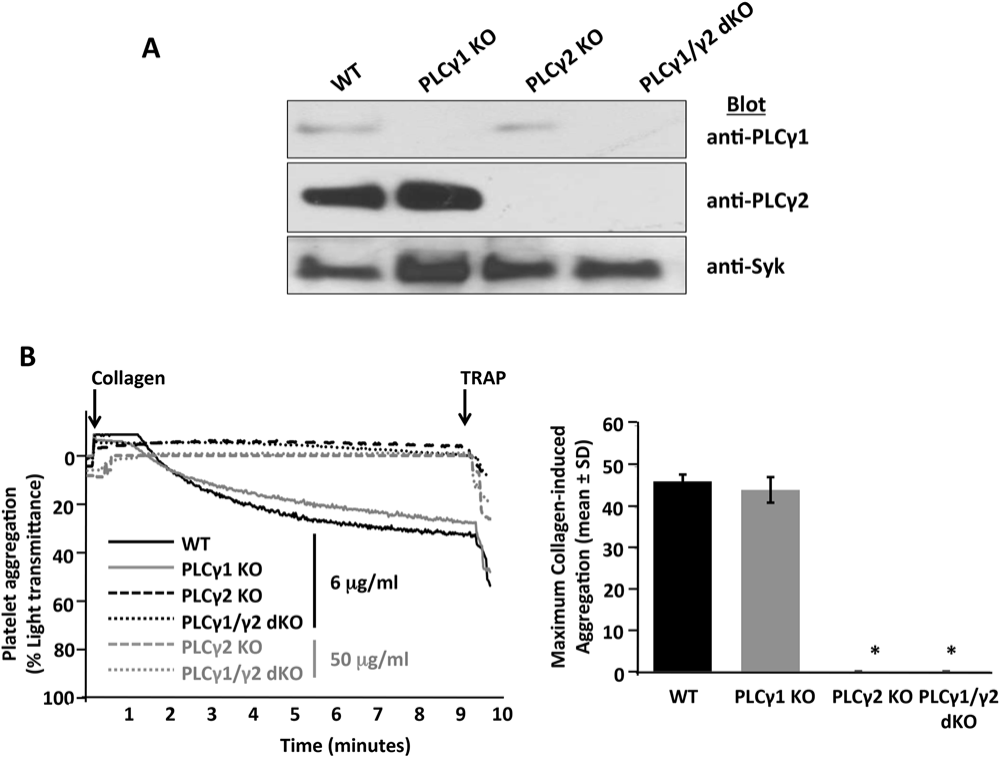
Effect of PLCγ1 or/and PLCγ2 deficiency on platelet aggregation in response to collagen stimulation. (A) Expression levels of PLCγ1 and PLCγ2 in platelets from wild-type (WT), PLCγ1-deficient (PLCγ1 KO), PLCγ2-deficient (PLCγ2 KO) and PLCγ1/γ2 double-deficient (PLCγ1/γ2 dKO) mice. Platelets were isolated from whole blood based on the standard platelet isolation protocol. Total cell lysates were used for direct Western blot analysis using antibodies specific for PLCγ1, PLCγ2 and Syk. (B) Washed platelets from WT, PLCγ1-deficient (PLCγ1 KO), PLCγ2-deficient (PLCγ2 KO) and PLCγ1/γ2 double-deficient (PLCγ1/γ2 dKO) mice were stimulated under stirring conditions with collagen at a final concentration of 6 μg/ml (black lines) or 50 μg/ml (gray lines). Thrombin receptor activating peptide (TRAP; 5 μg/ml) was added at 5 minutes as a positive control. Results were recorded on a Chrono-log Platelet Aggregometer. A representative aggregometry plot (adjusted for the timing of addition of collagen and TRAP) is shown on the left, and quantitative analysis of maximum aggregation induced by collagen (6 μg/ml) observed in three independent experiments is shown on the right (*p < 0.0001 relative to WT). Note that loss of PLCγ2 abrogates, but loss of PLCγ1 has no effect on, collagen-induced platelet aggregation.

PLCγ2 deficiency has previously been shown to dramatically impact platelet activation via the collagen-GPVI signaling pathway;[17–20] however, the effect of PLCγ1 deficiency on GPVI-induced platelet activation has not previously been evaluated. To investigate the role of PLCγ1 in collagen-induced platelet activation, we generated PLCγ1-deficient and PLCγ1/γ2 double-deficient mice and compared their responses to those of platelets derived from wild-type control and PLCγ2-deficient mice. Western blot analysis (Fig. 3A) verified that PLCγ1-deficient platelets expressed wild-type levels of PLCγ2 but no PLCγ1, PLCγ2-deficient platelets expressed wild-type levels of PLCγ1 but no PLCγ2 and PLCγ1/γ2 double-deficient platelets failed to express either PLCγ1 or PLCγ2. These results confirmed the specific depletion of the relevant PLCγ isoform(s) in the deficient mice, and also demonstrated that the absence of one of the PLCγ isoforms does not affect the level of expression of the other isoform. Platelet counts in PLCγ1- and/or PLCγ2-deficient mice were normal (data not shown), which indicates that PLCγ is not required for megakaryopoiesis or platelet maturation in mice. In addition, expression levels of relevant major platelet receptors, including GPVI, αIIbβ3, GPIb/V/IX and α2β1, were not affected by the absence of PLCγ1 and/or PLCγ2 (S1 Table), which suggests that PLCγ1 and PLCγ2 are not required for expression of these major platelet receptors.

To investigate the relative roles of PLCγ1 and PLCγ2 in collagen-induced platelet activation, platelets from wild-type, PLCγ1-deficient, PLCγ2-deficient and PLCγ1/γ2 double-deficient mice were isolated, and collagen-triggered platelet aggregation was examined. As a positive control, platelets were also stimulated with 5 μg/ml thrombin receptor activating peptide (TRAP), which activates platelets through a G protein-coupled receptor (GPCR) pathway that relies on PLCβ for generation of IP_3_ and DAG. As shown in Fig. 3B, platelets from PLCγ2-deficient and PLCγ1/γ2 double-deficient mice were unable to aggregate in response to low (6 μg/ml) or high concentrations (50 μg/ml) of collagen. In contrast, PLCγ1-deficient platelets aggregated to the same extent in response to collagen stimulation as did wild-type platelets (Fig. 3B). These data indicate that PLCγ2 is required for collagen-induced platelet aggregation and that PLCγ1 normally plays no role in this process.

**Fig 4:**
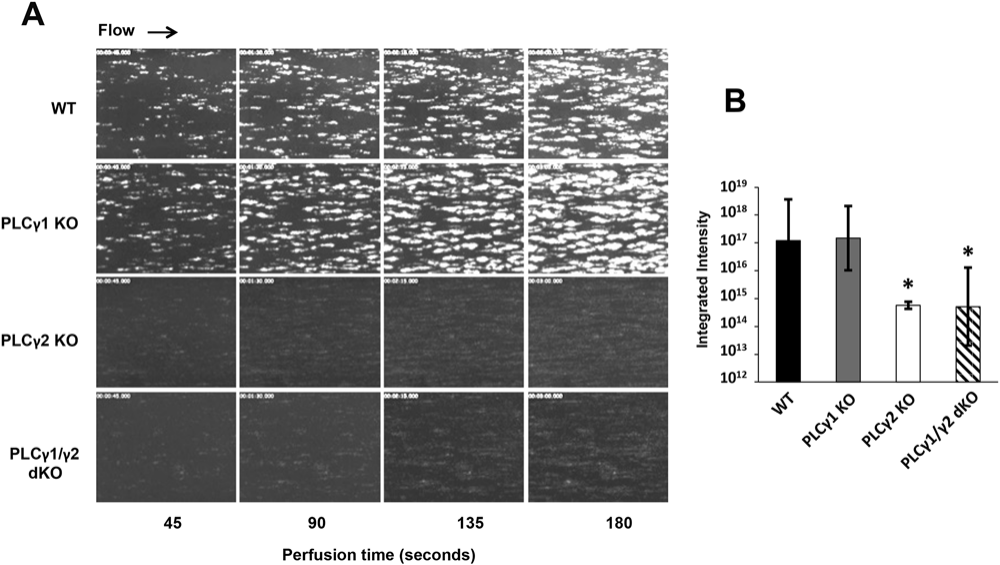
Effect of PLCγ1 or/and PLCγ2 deficiency on platelet thrombus formation on collagen. Laminar flow chambers were coated with 50 μg/ml of type I fibrillar collagen and blocked with Hank’s Balancing Salt Solution containing 0.1% BSA. Whole blood from WT, PLCγ1-deficient (PLCγ1 KO), PLCγ2-deficient (PLCγ2 KO) and PLCγ1/γ2 double-deficient (PLCγ1/γ2 dKO) mice was anticoagulated with heparin and PPACK, labeled with mepacrine, and perfused under conditions of arterial (1333s^-1^) shear. Images of platelet adhesion and accumulation were acquired using epifluorescence microscopy in real-time at a rate of one frame per second. (A) Representative images of platelet adhesion and accumulation over time. Data shown are representative of 3–4 independent experiments. (B) Platelet thrombi formed at 180 seconds in 3–4 independent experiments were quantified using MetaMorph software. Results are expressed as mean total integrated fluorescence intensity ± SD, which is presented on a log scale on the y-axis. Statistically significant differences between the means were determined using Student’s t test. Note that PLCγ1-deficient platelets formed thrombi normally on collagen-coated surfaces under conditions of shear stress, whereas PLCγ2-deficient and PLCγ1/γ2 double-deficient platelets formed significantly smaller thrombi (*p < 0.05 relative to WT).

Platelet thrombus formation on collagen-coated surfaces under conditions of arterial shear stress requires adhesion by the GPIb/V/IX complex to VWF and subsequent activation by the GPVI collagen receptor. Whereas PLCγ2 deficiency has previously been shown to diminish thrombus formation on collagen-coated surfaces,[28] the effect of PLCγ1 deficiency on thrombus formation has not previously been evaluated. We used a whole-blood microfluidic perfusion system to examine the relative contributions of PLCγ1 and PLCγ2 to platelet adhesion and thrombus formation on a fibrillar collagen-coated surface under conditions of arterial shear.[29] Platelets in whole blood were labeled with mepacrine, and accumulation of fluorescent platelets on collagen-coated surfaces was used to quantify adhesion and thrombus generation. As shown in Fig. 4, PLCγ1-deficient platelets formed thrombi that were comparable to those formed by wild-type platelets, whereas platelets from either PLCγ2-deficient or PLCγ1/γ2 double-deficient mice, which adhered to collagen-coated surfaces relatively normally (data not shown), failed to form thrombi. These data indicate that PLCγ2 is required for thrombus formation initiated by collagen, and that PLCγ1 plays little, if any, role in this process.

### Deficiency of PLCγ2, but not PLCγ1, abrogates αIIbβ3-mediated platelet spreading

**Fig 5:**
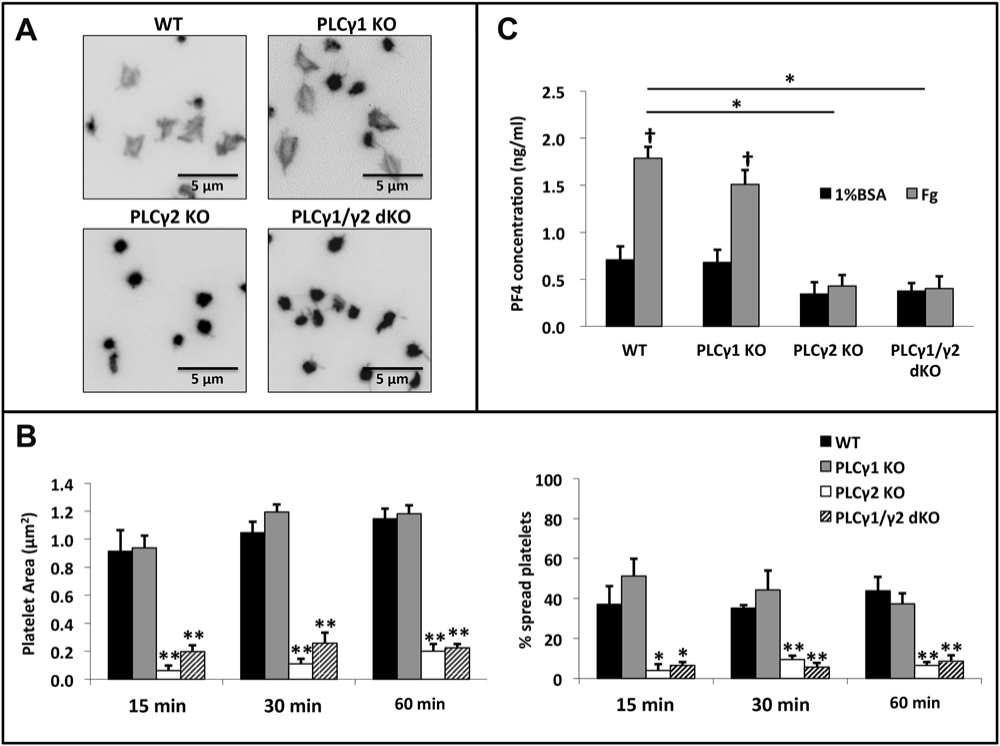
Effect of PLCγ1 or/and PLCγ2 deficiency on platelet spreading and granule secretion on immobilized Fg. (A) Washed platelets from WT, PLCγ1-deficient (PLCγ1 KO), PLCγ2-deficient (PLCγ2 KO) and PLCγ1/γ2 double-deficient (PLCγ1/γ2 dKO) mice were plated onto 8-chamber glass tissue-culture slides coated with Fg (3 μg/ml), and allowed to spread for up to 60 minutes at 37°C. Platelets were fixed, permeabilized, and stained for F-actin using TRITC-Phalloidin. (B) Quantitative analysis of mouse platelets spread on immobilized Fg. Platelet spreading was quantified using Metamorph software (for each genotype, at least 200 platelets were analyzed), platelet spreading area (μm^2^) and percentage (%) are shown. Results are reported as mean ± S.D. from 3 independent experiments using 3 different groups of mice. (C) Assessment of granule secretion from spread platelets. Supernatants of platelets allowed to spread on fibrinogen for 60 minutes were collected and assayed for PF4 concentration by ELISA. Results are reported as mean ± S.D. from 3 independent experiments (*p < 0.001, **p < 0.0001 for each genotype relative to wildtype; †p < 0.0005 for Fg relative to 1% BSA). Note that platelets from PLCγ2-deficient or PLCγ1/γ2 double-deficient mice showed a significant reduction in platelet spreading and granule secretion on immobilized Fg when compared to WT and PLCγ1-deficient platelets.

PLCγ2-deficient mice exhibit residual αIIbβ3-mediated platelet activation,[17–20] raising the possibility that PLCγ1 contributes to this process. To determine the relative contributions of PLCγ1 and PLCγ2 to platelet activation by αIIbβ3, we first examined the effect of PLCγ1 and/or PLCγ2 deficiency on the ability of platelets to spread on fibrinogen-coated surfaces. As shown in Fig. 5A, PLCγ1-deficient platelets spread to a similar extent as did wild-type platelets on immobilized fibrinogen whereas platelets from PLCγ2-deficient and PLCγ1/γ2 double-deficient mice failed to spread. Quantitative analysis of platelet spreading revealed that neither the area nor the percentage of spread platelets differed significantly between wild-type and PLCγ1-deficient platelets, whereas both the area and percentage of spread platelets from PLCγ2-deficient and PLCγ1/γ2 double-deficient mice were significantly reduced relative to that observed with platelets from wild-type mice (Fig. 5B). Platelet spreading on immobilized fibrinogen requires that soluble agonists, such as ADP secreted from platelet granules, bind to GPCRs that activate αIIbβ3 via an inside-out signaling process that relies on activation of PLCβ.[15, 30] To determine whether PLCγ2 is required for αIIbβ3-mediated secretion of platelet granule contents, we measured the concentrations of the platelet α-granule protein, platelet factor 4 (PF4), in releasates of wild-type, PLCγ1-deficient, PLCγ2-deficient or PLCγ1/γ2 double-deficient platelets following incubation on fibrinogen-coated surfaces for 60 minutes at room temperature.[31, 32] We found that both wild-type and PLCγ1-deficient platelets released PF4 normally, whereas PLCγ2-deficient and PLCγ1/γ2 double-deficient platelets did not secrete PF4, when allowed to spread on immobilized fibrinogen (Fig. 5C). These data indicate that PLCγ2 is required for αIIbβ3-dependent platelet spreading on fibrinogen, and that PLCγ1 plays no role in this process.

**Fig 6:**
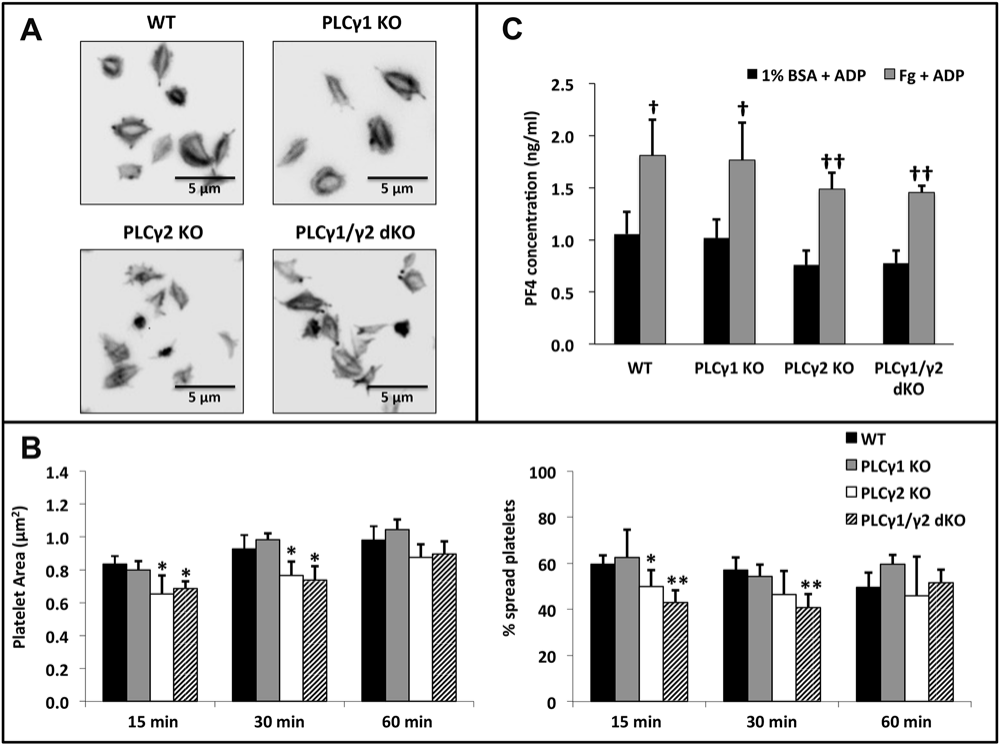
Effect of PLCγ1 or/and PLCγ2 deficiency on platelet spreading and granule secretion on immobilized Fg in the presence of ADP. Washed platelets from WT, PLCγ1-deficient (PLCγ1 KO), PLCγ2-deficient (PLCγ2 KO) and PLCγ1/γ2 double-deficient (PLCγ1/γ2 dKO) mice were allowed to spread on Fg (3 μg/ml) in the presence of 20 μM ADP for up to 60 minutes at 37°C. (A) Platelets were fixed, permeabilized, and stained for F-actin using TRITC-Phalloidin. (B) Quantitative analysis of mouse platelets spread on immobilized Fg. Platelet spreading was quantified using Metamorph software (for each genotype, at least 200 platelets were analyzed), platelet spreading area (μm^2^) and percentage (%) are shown. Results are reported as mean ± S.D. from 3 independent experiments using 3 different groups of mice. (C) Assessment of granule secretion from spread platelets. Supernatants of platelets allowed to spread on fibrinogen in the presence of ADP for 60 minutes were collected and assayed for PF4 concentration by ELISA. Results are reported as mean ± S.D. from 3 independent experiments (*p < 0.05, **p < 0.001 for each genotype relative to wildtype; †p < 0.05, ††p < 0.005 for Fg + ADP relative to 1% BSA + ADP). Note that the failure of PLCγ2-deficient or PLCγ1/γ2 double-deficient platelets to spread or release granule contents on immobilized Fg was overcome by addition of exogenous ADP.

To determine whether PLCγ2 is required only for granule secretion or if it is also required for αIIbβ3-mediated platelet spreading once αIIbβ3 has been activated in response to secreted agonists, we assessed the ability of exogenous ADP to restore spreading of PLCγ2-deficient and PLCγ1/γ2 double-deficient platelets on immobilized fibrinogen. PLCγ2-deficient and PLCγ1/γ2 double-deficient platelets spread on fibrinogen (Fig. 6A, B) and secreted granule contents (Fig. 6C) to the same extent as did wild-type and PLCγ1-deficient platelets after 60 minutes in the presence of ADP. The slightly but significantly lower levels of spreading exhibited by PLCγ2-deficient and PLCγ1/γ2 double-deficient relative to WT and PLCγ1-deficient platelets at earlier time points is consistent with a role for PLC2 in amplification of ADP-induced granule secretion and subsequent spreading of platelets on immobilized fibrinogen. Taken together, these data indicate that, in the process of αIIbβ3-dependent platelet spreading on fibrinogen, PLCγ2 is required for release of soluble agonists from platelet granules, which then bind to GPCRs and enable activation of αIIbβ3 so that it can support platelet spreading.

### Over-expression of PLCγ1 restores the defects in platelet activation caused by PLCγ2 deficiency

**Fig 7:**
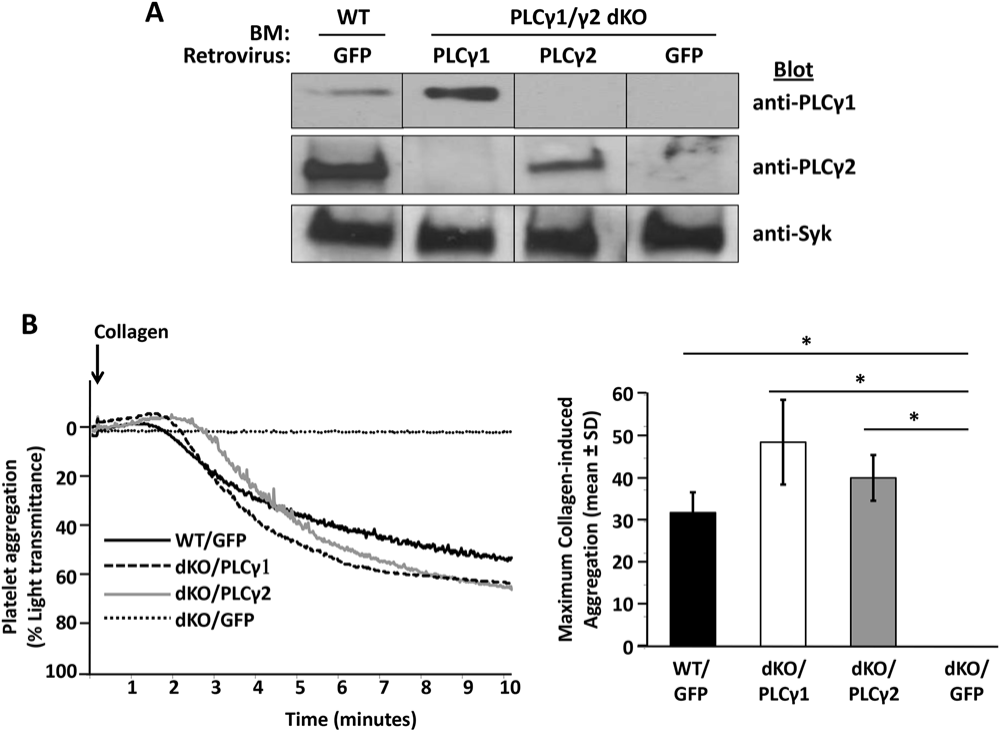
Restoration of collagen-induced platelet aggregation by enforced expression of PLCγ1 in PLCγ1/γ2 double-deficient platelets. Lethally irradiated wild-type (WT) mice were reconstituted with WT bone marrow transfected with IRES-GFP retroviruses (WT/GFP) or with PLCγ1/γ2 double-deficient bone marrow transfected with IRES-GFP retroviruses (dKO/GFP), PLCγ1-IRES-GFP retroviruses (dKO/PLCγ1), or PLCγ2-IRES-GFP retroviruses (dKO/PLCγ2). Recipient mice were analyzed 8 weeks after reconstitution. (A) Overexpression of PLCγ1 or PLCγ2 in PLCγ1/γ2 double-deficient platelets. Washed non-sorted platelets from reconstituted recipients were subjected to direct Western blot analysis with antibodies specific for PLCγ1, PLCγ2 and Syk. (B) Washed non-sorted platelets from reconstituted recipients were stimulated under stirring conditions with collagen (6 μg/ml). Results were recorded on a Chrono-log Platelet Aggregometer. A representative aggregometry plot (adjusted for the timing of addition of collagen) is shown on the left, and quantitative analysis of the results of two independent experiments is shown on the right (*p < 0.005 for each genotype relative to dKO/GFP). Note that defective collagen-induced platelet aggregation was overcome by enforced expression of either PLCγ1 or PLCγ2 in PLCγ1/γ2 double-deficient platelets.

Collectively, the data presented thus far demonstrate that PLCγ2 is required for platelet activation by GPVI and αIIbβ3, and that PLC1 normally plays no role in signal transduction by these receptors. To determine whether the low levels at which PLCγ1 is normally expressed are responsible for its inability to participate in platelet activation, we used a strategy involving retrovirus-mediated gene transfer and bone marrow reconstitution to generate mice with platelets that overexpressed PLCγ1.[26] PLCγ1/γ2 double-deficient bone marrow cells were transduced *in vitro* with a retrovirus encoding PLCγ1, an internal ribosome entry site (IRES), and green fluorescent protein (GFP). PLCγ1/γ2 double-deficient bone marrow cells were also transduced with a retrovirus encoding IRES-GFP alone as a negative control, or with a retrovirus encoding PLCγ2-IRES-GFP as a positive control. As a second positive control, bone marrow cells from wild-type mice were transduced with a retrovirus encoding GFP. Transduced bone marrow cells were transplanted into lethally irradiated C57BL/6 wild-type mice. Following reconstitution, platelets from the recipients were analyzed for GFP positivity by flow cytometry to determine transduction efficiency. 11% of platelets were GFP-positive in mice reconstituted with IRES-GFP-transduced wild-type bone marrow, and the percent of GFP-positive platelets in mice reconstituted with retrovirally transduced PLCγ1/γ2 double-deficient bone marrow was 32% for PLCγ1-IRES-GFP, 49% for PLCγ2-IRES-GFP, and 29% for IRES-GFP. Levels of expression of PLCγ1 and PLCγ2 in platelets obtained from reconstituted mice were determined by Western blot analysis. As shown in Fig. 7A, transduction of PLCγ1/γ2 double-deficient platelets with the PLCγ1-encoding retrovirus resulted in much higher levels of expression of PLC1 than were observed in wild-type platelets, whereas PLC2-transduced PLCγ1/γ2 double-deficient platelets expressed PLCγ2 at a level that was slightly lower than that observed in wild-type platelets. To quantify the relative levels of PLCγ1 and PLCγ2 in platelets obtained from reconstituted mice, increasing amounts of undiluted or 1:70 diluted lysates of highly purified mouse platelets were subjected to Western blot analysis with antibodies specific for PLCγ1 or PLCγ2, respectively. As shown in S1 Fig, densitometric analysis of the PLCγ1/PLCγ2 blots of platelet lysates revealed that levels of PLCγ1 in platelets from PLCγ1/γ2 double-deficient mice reconstituted with PLCγ1 retrovirus-transduced bone marrow (32% of which were GFP- and presumably PLCγ1-positive) were approximately 140 times higher than the levels at which endogenous PLCγ1was expressed in wild-type platelets (e.g., the PLCγ1 band intensity in 20 μl of undiluted wild-type platelet lysate was equivalent to the PLCγ1 band intensity observed with 10 μl of 1:70 diluted PLCγ1-reconstituted platelet lysate). Levels of PLCγ2 in platelets from PLCγ1/γ2 double-deficient mice reconstituted with PLCγ2 retrovirus-transduced bone marrow (49% of which were GFP- and presumably PLCγ2-positive), in contrast, were equivalent to the levels at which endogenous PLCγ2 was expressed in wild-type platelets (i.e., the PLCγ2 band intensity of 1:70 diluted wild-type platelet lysate was within ~70–80% that of the PLCγ2 band intensity of 1:70 diluted PLCγ2-reconstituted platelet lysate). Finally, the finding that similar amounts of platelet lysate were required to achieve a PLCγ1 band intensity equivalent to that of PLCγ2 (e.g., the PLCγ1 band intensity in 10 μl of 1:70 diluted PLCγ1-reconstituted platelet lysate was equivalent to the PLCγ2 band intensity observed with 10 μl of 1:70 diluted PLCγ2-reconstituted or wild-type platelet lysate), together with the finding that anti-PLCγ1 recognizes PLCγ1 approximately 3 times better than anti-PLCγ2 recognizes PLCγ2 (see Fig. 1, above), indicates that the level at which PLCγ1 was expressed in PLCγ1-reconstituted platelets was much closer (3X rather than 400X less) to the level at which PLCγ2 was expressed in either wild-type or PLCγ2-reconstituted platelets.

**Fig 8:**
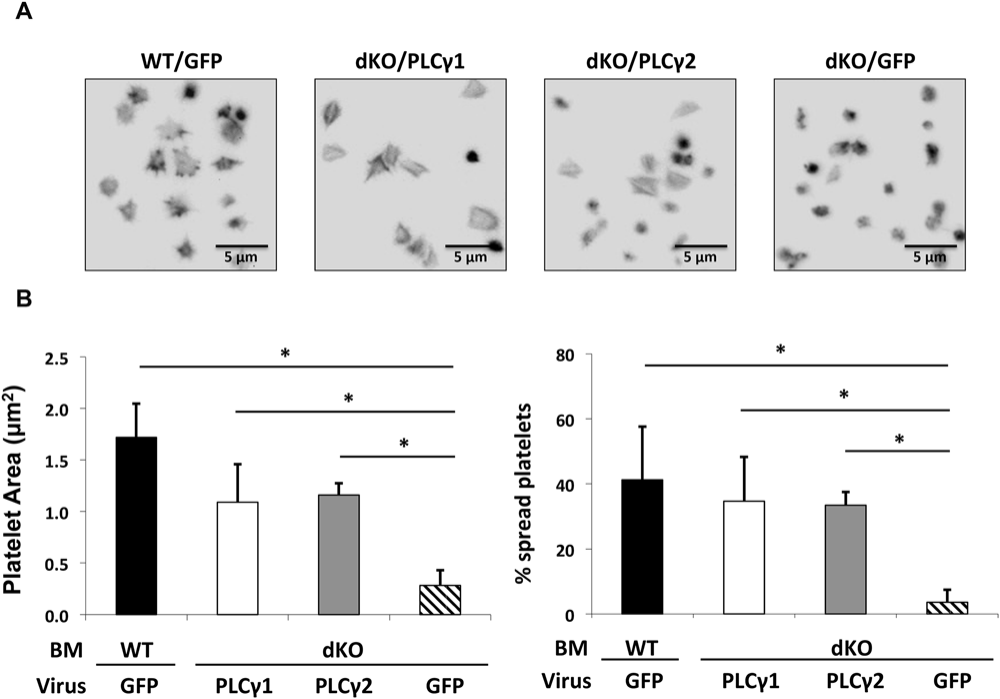
Restoration of αIIbβ3-mediated platelet spreading on Fg by enforced expression of PLCγ1 or PLCγ2 in PLCγ1/γ2 double-deficient platelets. Lethally irradiated wild-type (WT) mice were reconstituted with WT bone marrow transfected with IRES-GFP retroviruses (WT/GFP) or with PLCγ1/γ2 double-deficient bone marrow transfected with IRES-GFP retroviruses (dKO/GFP), PLCγ1-IRES-GFP retroviruses (dKO/PLCγ1), or PLCγ2-IRES-GFP retroviruses (dKO/PLCγ2). Recipient mice were analyzed 8 weeks after reconstitution. (A) Washed non-sorted platelets from reconstituted recipients were allowed to spread on Fg (3 μg/ml) for 60 minutes at 37°C. Platelets were fixed, permeabilized, and stained for F-actin using TRITC-Phalloidin. (B) Quantitative analysis of mouse platelet spreading on immobilized Fg. Platelet spreading was quantified using Metamorph software (for each genotype, at least 300 platelets were analyzed), platelet spreading area (μm^2^) and percentage (%) are shown. Quantitative analysis was performed on all non-sorted platelets regardless of GFP positivity. Results are reported as mean ± S.D. from 2 independent experiments (*p < 0.05 for each genotype relative to dKO/GFP). Note that increased platelet spreading area and spreading percentage were observed in PLCγ1/γ2 double-deficient platelets that expressed PLCγ2 or PLCγ1 at levels normally achieved by PLCγ2.

To determine whether PLCγ1, when expressed at levels only 3 times less than that of PLCγ2 in WT platelets, can support platelet activation by GPVI and αIIbβ3, we examined the ability of reconstituted platelets, which represent a heterogeneous population of transduced and non-transduced platelets, to aggregate in response to collagen stimulation and spread on immobilized fibrinogen. As shown in Fig. 7B, whereas platelets from mice reconstituted with IRES-GFP-transduced PLCγ1/γ2-deficient bone marrow (dKO/GFP) failed to aggregate in response to collagen stimulation, platelets from mice reconstituted with PLCγ1-transduced PLCγ1/γ2-deficient bone marrow (dKO/PLCγ1) aggregated to a similar extent as did platelets from mice reconstituted with GFP-transduced WT bone marrow (WT/GFP) or with PLCγ2-transduced PLCγ1/γ2-deficient bone marrow (dKO/PLCγ2). Similarly, as shown in Fig. 8, whereas platelets from mice reconstituted with GFP-transduced PLCγ1/γ2-deficient bone marrow (dKO/GFP) failed to spread on immobilized fibrinogen, platelets from mice reconstituted with PLCγ1-transduced PLCγ1/γ2-deficient bone marrow (dKO/PLCγ1) spread to the same extent as did platelets from mice reconstituted with GFP-transduced WT bone marrow (WT/GFP) or with PLCγ2-transduced PLCγ1/γ2-deficient bone marrow (dKO/PLCγ2). The homogenous spreading response of dKO/PLCγ1 and dKO/PLCγ2 platelets, despite transduction efficiencies of only 32% for 49% for PLCγ1- and PLCγ2-reconstituted mice, respectively, is likely explained by a paracrine effect of ADP released from the successfully transduced platelets on the non-transduced platelet population. Taken together, these data demonstrate that, when expressed at sufficiently high levels, PLCγ1 can fully support GPVI- and αIIbβ3-dependent platelet responses.

## Discussion

In this report, we use PLCγ1-deficient, PLCγ2-deficient, and PLCγ1/2-double deficient mice to elucidate the roles of PLCγ1 and PLCγ2 in platelet activation. Our findings confirm previous reports that PLCγ2 is required for collagen/GPVI-mediated platelet aggregation, GPVI-dependent thrombus formation on collagen under conditions of shear, and αIIbβ3-mediated platelet spreading on immobilized fibrinogen.[17–20, 22–24] Moreover, PLCγ2 is indispensable for platelet granule secretion downstream of adhesive interactions mediated by αIIbβ3, which is required for spreading on fibrinogen. Addition of exogenous ADP rescues defective spreading of PLCγ2-deficient platelets on fibrinogen, which indicates that absence of PLCγ2 does not affect the function of the spreading machinery. Our studies also reveal that mouse platelets contain approximately 400 times more PLCγ2 than PLC1, and that expression of PLC1 at levels normally achieved by PLCγ2 can fully restore both GPVI-dependent aggregation and αIIbβ3-dependent spreading in platelets that lack PLCγ2. Taken together, these data demonstrate that platelet activation by GPVI and αIIbβ3 normally requires PLCγ2, but that PLCγ1, which is normally expressed at levels that are insufficient to support these processes, can fully support platelet activation if it is expressed at sufficiently high levels.

Our findings are completely compatible with results of previous studies demonstrating that PLCγ2-deficiency abrogated platelet responses to GPVI-specific stimuli and diminished platelet thrombus formation on von Willebrand factor under flow conditions.[14, 17–20, 28] However, our results contrast with previous reports that PLCγ2-deficient platelets were only partially impaired in their ability to retract a fibrin clot or to fully spread on immobilized fibrinogen[22–24] and that, whereas PLCγ2-deficient platelets failed to respond to GPVI-specific agonists, they were able to mount responses to collagen[17, 33]. The partial responses of PLCγ2-deficient platelets to fibrinogen observed in previous studies may have been due to the presence of trace amounts of GPCR agonists, which we found to be able to bypass the need for PLCγ2 in αIIbβ3-dependent activation of platelets. The explanation for differences in the extent to which we and others detected responses of PLCγ2-deficient platelets to collagen is not known, but may be attributable to the different sources of collagen used to stimulate platelets. Our findings that PLCγ2-deficiency abrogated, and PLCγ1 deficiency had no effect on, either platelet spreading on fibrinogen or thrombus formation on collagen under conditions of shear definitively demonstrate that PLCγ1 normally plays no role in platelet activation by either fibrinogen or collagen.

Although PLCγ1 and PLCγ2 play critical roles in the responsiveness of hematopoietic cells to stimulation via immune receptors,[9] hematopoietic cells differ in the extent to which the rely on PLCγ isoforms for development. PLCγ1 is the predominant PLCγ isoform expressed in T cells, and is required for T cell development, activation and tolerance.[25] In B cells, the key signaling component downstream of the BCR is PLCγ2, which plays essential roles in B cell development, differentiation and function.[13, 14] Both PLCγ1 and PLCγ2 contribute to the maturation of B and T lymphocytes [26, 34, 35] and, although controversial, possibly also to the maturation of NK cells.[36, 37] Interestingly, PLCγ2 is highly expressed only at early stages of T cell maturation, whereas PLCγ1 is expressed at all stages of T cell development.[34] Similarly, during B cell development, the PLCγ1 isoform is highly expressed at the pro/pre-B cell stage and starts to decline in maturing B cells, whereas PLCγ2 expression levels remain constant at all developmental stages.[26] Together, these findings suggest that the presence of both PLCγ isoforms may be indispensable for the pre-BCR or pre-TCR to generate a sufficient amount of total PLC activity to drive maturation forward. Once the mature BCR or TCR is expressed on the cell surface, the major PLC isoform appears to be sufficient to enable responsiveness of mature cells. Like B cells, platelets use PLCγ2 as a principal signaling component in response to agonist stimulation;[19, 20, 22] however, unlike B cells, platelets require neither PLC1 nor PLCγ2 for development, since platelets develop normally in the absence of PLC2 alone [14] and in the absence of both PLC1 and PLCγ2 (present study). Other hematopoietic cells that develop normally in the absence of their major PLC isoform (PLC2) include neutrophils and macrophages.[14] The molecular mechanisms underlying the differential requirement of hematopoietic cells for PLCγ activity during development remains to be determined.

Several types of hematopoietic cells, including bone marrow derived macrophages (BMDM),[38] dendritic cells,[39] natural killer (NK) cells,[36, 37, 40] mast cells,[14] and neutrophils,[41, 42] are like platelets in that they normally rely solely on PLC2 for signal transduction downstream of ITAM-coupled receptors. Our findings that PLCγ2 is 400X more abundant than is PLCγ1 in platelets, and that over-expression of PLCγ1 completely restores responsiveness of PLCγ2-deficient or PLC1/γ2 double-deficient platelets indicates that PLCγ1 normally plays no role in platelet activation simply because its expression is limiting. If it is expressed at sufficiently high levels, PLCγ1 can fully compensate for PLC2 in platelets. This finding in platelets is different from those observed in B cell and NK cells, wherein PLCγ1 was only partially able to compensate for PLCγ2 deficiency.[26, 37] Thus, enforced expression of PLCγ1 in PLCγ2-deficient mice could restore Ca^2+^ flux in B cells but not B cell proliferation and development (22). Similarly, PLCγ1 over-expression rescued expression of Ly49 receptors during late stages of maturation, restored cytotoxicity but not to wild-type levels, and failed to rescue interferon production by NK cells.[37] One possible explanation for this difference is that PLCγ1 and PLCγ2 play redundant roles with respect to some functions, but distinct roles with respect to other functions. We found that, in platelets, PLC activity is required for secretion of granule contents, upon which subsequent platelet functions such as spreading and thrombus formation depend, and that PLCγ1 and PLCγ2 are redundant with respect to their ability to induce platelet granule secretion. Whereas certain NK cell effector functions, e.g., cytotoxicity, depend on granule release, others rely on synthesis of cytokines, which requires initiation of transcription and translation. It is possible that PLCγ1 and PLCγ2 play redundant roles in induction of granule release, but that PLCγ2 functions uniquely with respect to transcription initiation. Additional studies of the ability of PLCγ1 over-expression to restore responsiveness of PLCγ1/γ2 double-deficient cells are needed to determine the extent to which PLCγ1 and PLCγ2 play redundant vs. unique roles in the different functions attributable to platelets, NK cells, and other PLCγ2-reliant hematopoietic cells. Although PLCγ1 and PLCγ2 are similar with respect to domain composition and overall conformation they are only 52% identical at the amino acid level, suggesting that the inter- and intra-molecular interactions that regulate their activity may be quite different. To the extent that unique functions for PLCγ2 are identified, studies of chimeric forms of PLCγ1 and PLCγ2 will be necessary to determine the distinct roles of different components of each PLCγ isoform in regulating signal transduction in different hematopoietic cell types.

## Supporting Information

S1 Fig Quantification of relative levels of PLCγ1 and PLCγ2 in retrovirus-transduced PLCγ1/γ2 double-deficient platelets. Increasing amounts of undiluted or 1:70 diluted highly purified mouse platelet lysate were subjected to Western blot analysis with antibodies specific for PLCγ1 (A) or PLCγ2 (B). Numbers under each lane indicate the density of each band. Note that levels of over-expressed PLCγ1 in PLCγ1-encoding retrovirus-transduced PLCγ1/γ2 double-deficient platelets were approximately 140 times more than endogenous PLCγ1 in wild-type platelets (A). Levels of over-expressed PLCγ2 in PLCγ2-encoding retrovirus-transduced PLCγ1/γ2 double-deficient platelets were approximately 2 times less than endogenous PLCγ2 in wild-type platelets (B).
(TIF)

S1 Table Comparison of major platelet receptors among platelets isolated from wild-type, PLCγ1-deficient, PLCγ2-deficient and PLCγ1/γ2 double-deficient mice. (TIF)
