# Supplementary figures and images for: Restoration of Responsiveness of Phospholipase Cγ2-Deficient Platelets by Enforced Expression of Phospholipase Cγ1
Source: PLoS One. 2015 Mar 20;10(3):e0119739. doi: 10.1371/journal.pone.0119739 (PMC4368822; doi:10.1371/journal.pone.0119739)

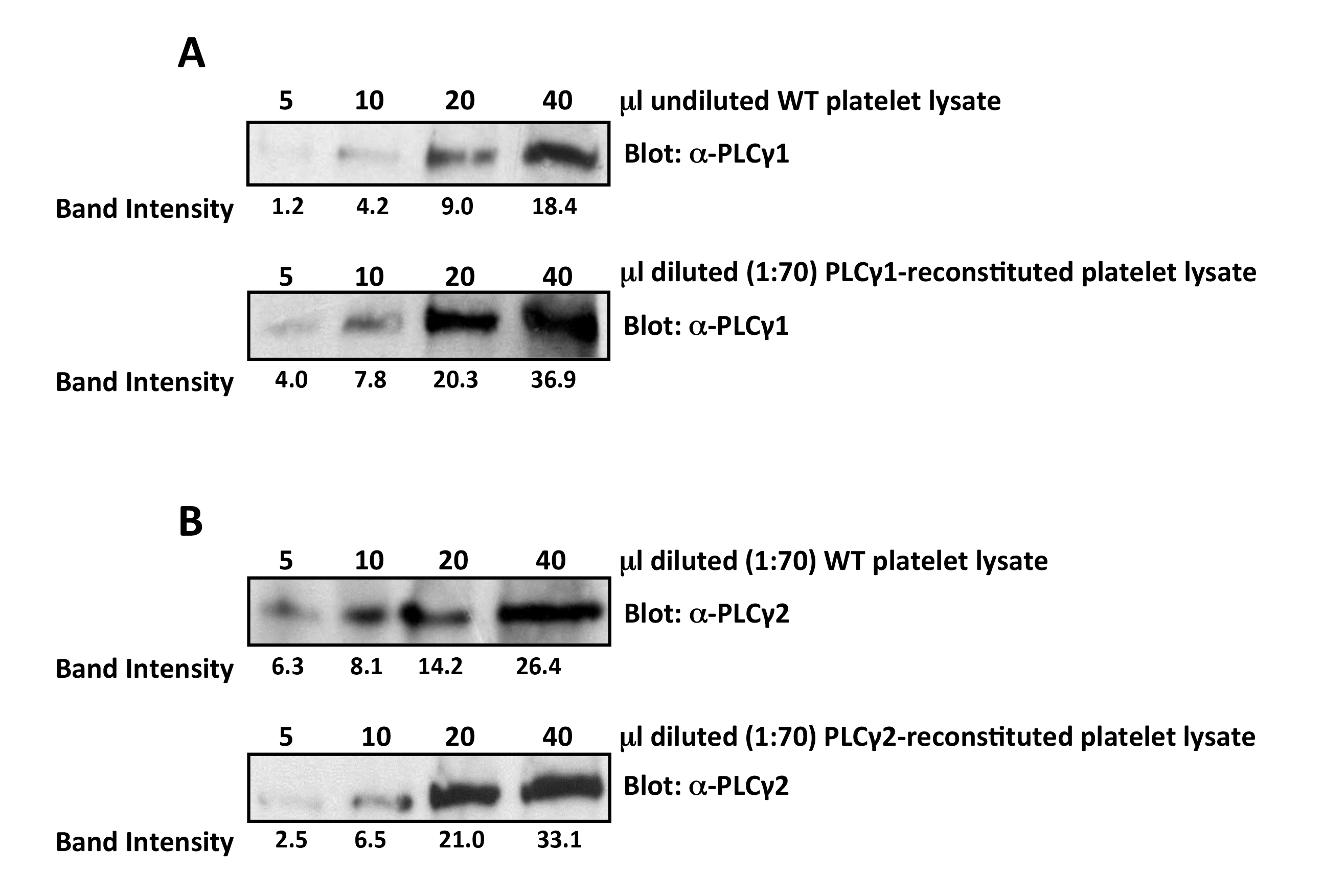

Supplement: S1 Fig — Increasing amounts of undiluted or 1:70 diluted highly purified mouse platelet lysate were subjected to Western blot analysis with antibodies specific for PLCγ1 (A) or PLCγ2 (B). Numbers under each lane indicate the density of each band. Note that levels of over-expressed PLCγ1 in PLCγ1-encoding retrovirus-transduced PLCγ1/γ2 double-deficient platelets were approximately 140 times more than endogenous PLCγ1 in wild-type platelets (A). Levels of over-expressed PLCγ2 in PLCγ2-encoding retrovirus-transduced PLCγ1/γ2 double-deficient platelets were approximately 2 times less than endogenous PLCγ2 in wild-type platelets (B). (TIF) [file pone.0119739.s001.tif]

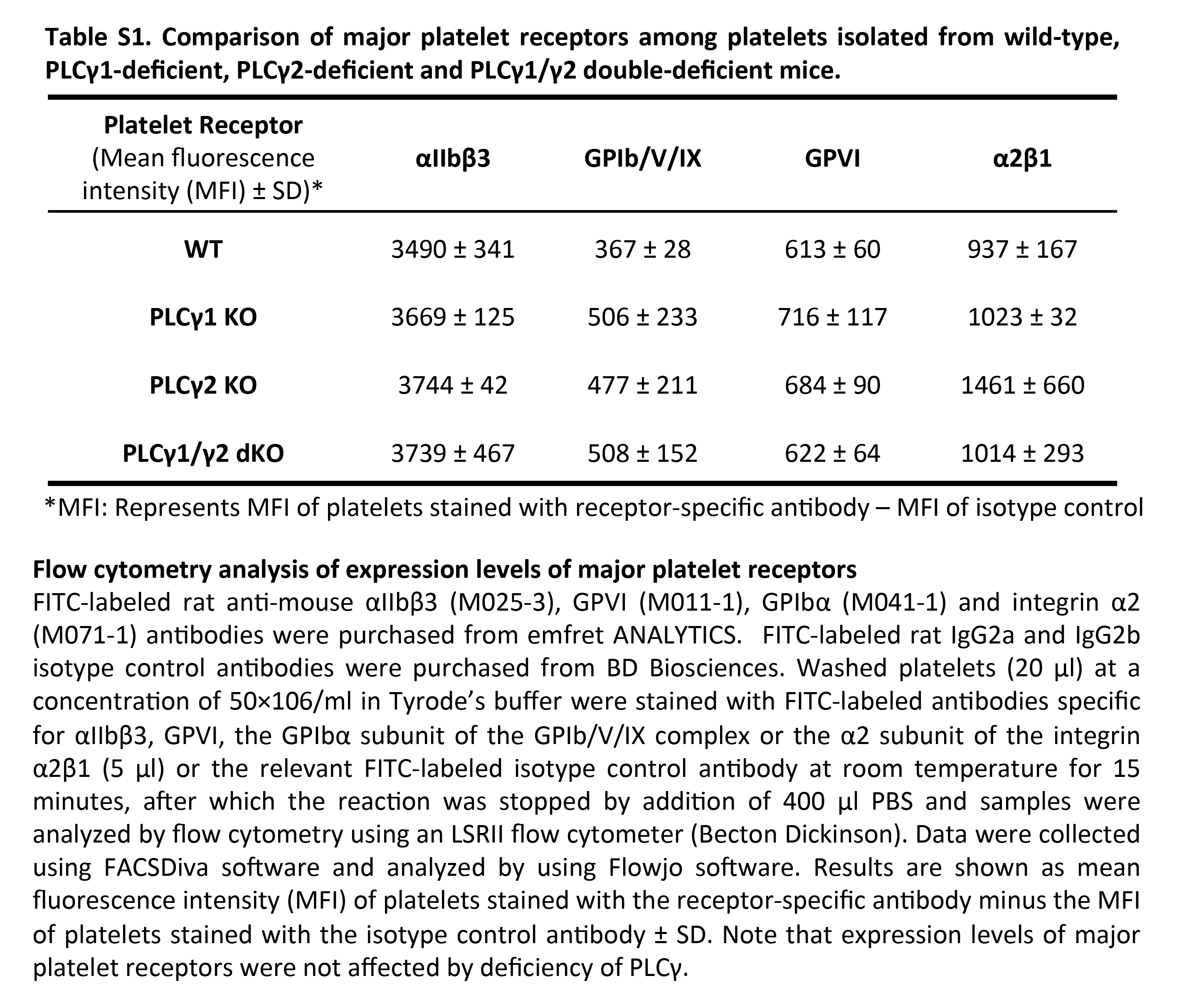

Supplement: S1 Table — (TIF) [file pone.0119739.s002.tif]
